# T1D REACHOUT–A Mobile App to Deliver Peer-Led Mental Health Support to Adults Living With Type 1 Diabetes: Co-Design and Development Process

**DOI:** 10.2196/71733

**Published:** 2026-03-20

**Authors:** Sadie Lee, Baray Sidhu, Parteek Johal, Ayman Azhar, Jonath Sujan, Matthias Görges, Tricia S Tang

**Affiliations:** 1 Faculty of Arts, University of British Columbia, Vancouver, BC, Canada; 2 Faculty of Science, University of British Columbia, Vancouver, BC, Canada; 3 Division of Respiratory Medicine, Department of Medicine, University of British Columbia, Vancouver, BC, Canada; 4 Department of Computer Science, University of British Columbia, Vancouver, BC, Canada; 5 AI and Data Science Unit, Research Institute, BC Children's Hospital, Rm V3-324, 950 West 28th Avenue, Vancouver, BC, V5Z 4H4, Canada, 1 604-875-2000 ext 5616; 6 Department of Anesthesiology, Pharmacology and Therapeutics, University of British Columbia, Vancouver, BC, Canada; 7 Division of Endocrinology, Department of Medicine, University of British Columbia, Vancouver, BC, Canada

**Keywords:** peer support, type 1 diabetes, digital interventions, user-centered design, mobile phone, mobile health, mHealth, app development

## Abstract

For adults living with type 1 diabetes (T1D), diabetes-specific mental health support is limited. Peer support and digital health platforms are promising strategies for delivering this support to this population, particularly those from geographically marginalized communities. Mobile apps, in particular, can enhance self-management and deliver support. This paper describes the iterative co-design and development process of a novel mobile app for use in a pilot trial, T1D REACHOUT (REACHOUT), that aims to reduce the diabetes distress, a core facet of diabetes-specific mental health, of adults with T1D. An initial think tank and 6 focus groups were conducted with adults with T1D to better understand their support needs and identify platform requirements. Following this, we partnered with adults living with T1D, the “end users,” to iteratively co-design the REACHOUT app, enhancing usability and ensuring relevance. Adapting the open-source Rocket.Chat platform to user-defined requirements, we deployed the app in a single cohort pilot study. A network analysis of messages exchanged during the pilot study was performed to explore trends and patterns and demonstrate implementation feasibility. Pilot study outcomes informed further refinement before implementation in a randomized controlled trial. The implementation of the REACHOUT app features 6 key components identified in 6 initial focus groups: a 24/7 chatroom (a customized group messaging function with threads), topic-specific discussion boards, a peer supporter library, peer supporter profiles for a user-driven matching process, small group virtual sessions, and direct (one-to-one) messaging. Forty-six participants were encouraged to use any or all of the features as frequently as desired over a 6-month period during the pilot study. During this time, 179 private small groups were created, and 10,410 messages were sent, including 1389 chat room messages and 7116 direct messages; among these were 3446 messages exchanged between participants and their self-selected peer supporters. Key factors for successful implementation included (1) the co-design process involving comprehensive user engagement and (2) the opportunities realized through hybrid development. These findings offer generalizable lessons for mobile health research teams developing similar app-based interventions.

## Introduction

### Mobile Apps for Type 1 Diabetes Management

In the era of digital health, mobile apps have increasingly been used to improve diabetes care. These platforms are vital for individuals living in geographically marginalized settings with limited access to conventional, in-person diabetes support [1]. Particularly, adults with type 1 diabetes (T1D) living in rural and remote communities in British Columbia have reported that peers with T1D share first-hand lived experience [1,2], from which they may benefit. As such, digitally delivered mental health support may be a promising solution for underserved populations.

Several systematic reviews and meta-analyses involving children or adults with T1D have examined mobile apps [3-7] designed to improve self-management [8-10], monitor treatment through data visualization [11], and deliver psychosocial interventions [12-15]. Notably, research in this area has mainly focused on mobile app evaluation, while descriptions of app development lag behind.

### Benefits of Involving End Users in App Development

Digital platforms that engage end users early in the development process are more likely to produce favorable results [16]. Specifically, co-design is a research methodology that, in principle, involves shared decision-making through the collaborative engagement of end users, researchers, and health care professionals to create and develop the technology to be used [17,18]. Thus, research exploring the mobile app preferences and perspectives of individuals with T1D has grown steadily [1,19,20]. Not surprisingly, 2 of the 4 best practices in app development involve “prioritizing user preferences” and “engaging stakeholders” [21].

While there is increased interest in collecting user feedback, how these data are integrated during the app development process has not been clearly described. Although several other T1D-specific apps provide detailed descriptions of their development, their goals are to improve glycated hemoglobin and blood glucose management by promoting self-care practices (carbohydrate counting, injection site rotation, insulin dosing, physical activity, etc) [22-25]. To our knowledge, no studies describe the comprehensive development process for a mobile app focused on reducing diabetes distress by offering peer-led mental health support to adults with T1D.

### The T1D REACHOUT Intervention

T1D REACHOUT (REACHOUT) is a mobile app designed to reduce diabetes distress by offering peer-led mental health support to adults with T1D living in rural and remote areas of Interior British Columbia [2]. Based on self-determination theory, which highlights what motivates patients to make support-related decisions [26], REACHOUT is a 6-month intervention that offers a range of support delivery modalities to meet users’ individual needs.

The use of the app is not intended to replace standard health care provision but rather to enable increased access to peer-led support and fill a gap in the health care system. While interdisciplinary diabetes care typically involves a team of endocrinologists, diabetes nurses, and dietitians, what is often overlooked and undervalued are the peers who understand first-hand the day-to-day experience of living with T1D.

### Aims

This paper describes the iterative co-design and development process of the REACHOUT mobile app, including its use in a pilot trial and subsequent revisions made prior to its implementation in a randomized controlled trial (RCT). While empirical outcomes from the REACHOUT pilot intervention have been reported elsewhere [2], we focus specifically on the technical development and implementation of the app used in the intervention and aim to share generalizable lessons with other mobile health (mHealth) research teams.

Specifically, this paper reflects on the (1) collaboration process between researchers and end users (ie, adults with T1D) to identify information needs and support requirements that informed app functionality, (2) iterative design of the app with end users to enhance usability and meet key user needs, (3) deployment of the app in a pilot study to understand feasibility, and (4) incorporation of feedback from the pilot study to further refine the app before launching the RCT.

This paper is organized in the following sections: Ethical Considerations; Recruitment; Platform Design and Implementation, which includes all stages of the co-design process; Platform Features, which outlines the functionality of the designed app; Initial Platform Utilization Patterns, which includes a network analysis of app usage during the pilot study; Insights and Observations, which specifically addresses challenges to co-design in mHealth; and Discussion, which includes an overview of our findings, limitations, and future work.

## Ethical Considerations

The University of British Columbia Behavioural Research Ethics Board approved this project (H20-00276, PI Tang, date of first approval 2020-03-31), with additional harmonized review by the Interior Health Research Ethics Board (same approval number). Participants were given study information, allowed to ask questions, and provided electronic consent. Focus group participants received a US $50 gift card upon completion. Pilot study participants received a US $25 and a US $50 Amazon gift card at baseline and at 6 months, respectively. Focus group discussions throughout the co-design process were recorded, transcribed verbatim, and then de-identified. Study data were stored securely with access limited to study team members.

## Recruitment

For our preliminary think tank session, we recruited participants (n=17) from a list of adults with T1D who expressed interest in T1D-specific mental health support at a Diabetes Canada-sponsored T1D patient advocacy event.

Focus group participants (n=38) were recruited using several modalities: (1) inviting participants from previous T1D diabetes studies, (2) obtaining referrals from health care providers, (3) sending emails to members of Breakthrough T1D living in the Interior British Columbia regions, (4) posting messages on T1D-specific social media sites, and (5) word of mouth [1]. Eligibility criteria included being diagnosed with T1D, ≥19 years, living in the Interior Health Authority region, and able to speak English [1].

Pilot study participants (n=46) were also identified and recruited via (1) diabetes education centers in the Interior Health Authority region, (2) T1D advocacy organizations, (3) T1D-specific Facebook support groups and social media outlets, (4) referrals from health care providers, and (5) word of mouth [2]. Eligibility criteria were similar to those of the focus group study, except that participants also needed to have access to the internet and/or a smartphone [2].

End user partners (n=11) were adults living with T1D who contributed to the iterative development process and were recruited from participants in the previous focus groups.

## Platform Design and Implementation

### Overview

**Figure 1.**
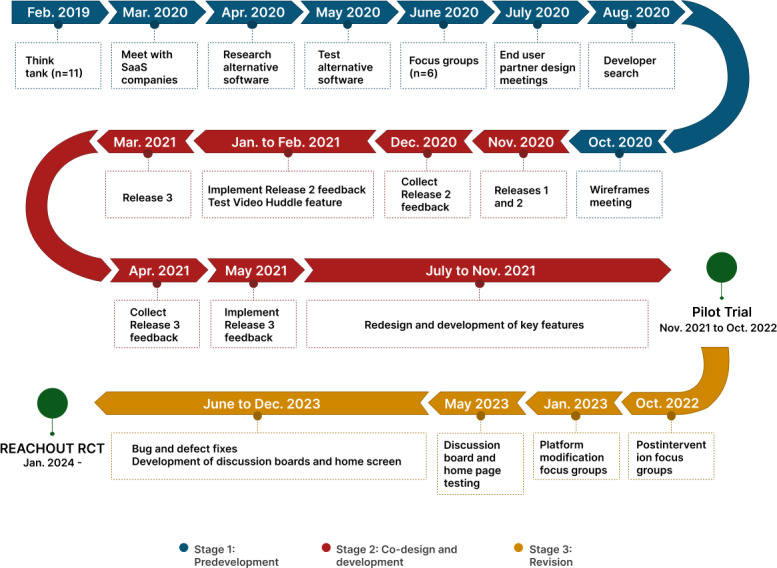
Timeline of the REACHOUT mobile app development process. Stage 1: predevelopment is indicated by blue arrows; stage 2: co-design and development are indicated by red arrows; and stage 3: deployment and revision are indicated by yellow arrows. Study milestones, including the pilot trial and the randomized controlled trial (RCT), are indicated by green circles. SaaS: software as a service.

**Figure 2.**
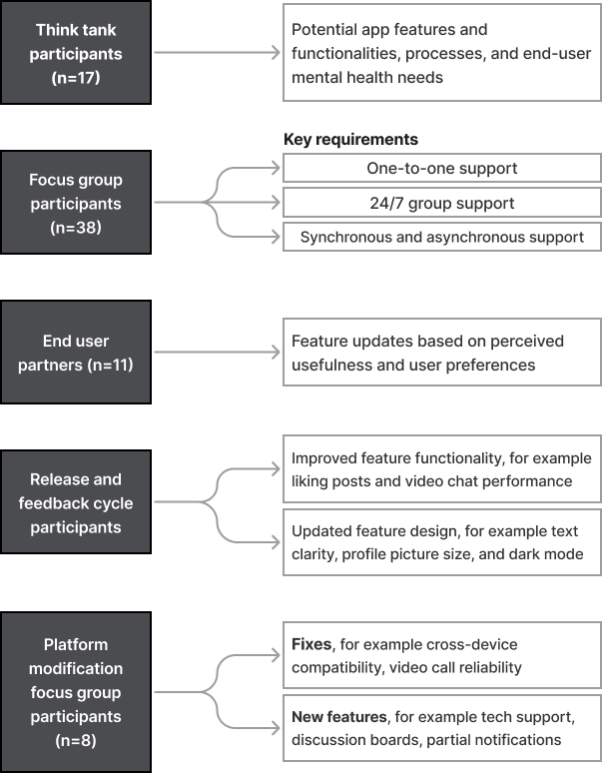
Co-design and development process, with each of the user groups engaged and the actions performed as a result of the activity.

Our development process occurred in 3 stages: predevelopment, co-design and development, and revision (Figure 1). As people with T1D are the end users of the REACHOUT app, their involvement was imperative; hence, their feedback and guidance were sought throughout the app’s development process to ensure it met end user needs. Through focus groups, informal think tanks, and feedback sessions, the co-design approach defined requirements and critical features for the peer support–based mobile app (Figure 2).

### Stage 1: Predevelopment

#### Think Tank

A preliminary think tank of adults with T1D was used as a scoping activity to elicit the support needs of this group and explore the possibility of a digital platform as the modality for emotional support. The session lasted approximately 60 minutes and was audio-recorded, with only an audio transcript available. Insights from this discussion informed the subsequent pursuit of a mobile app–based intervention and the continued use of a co-design approach.

Participants identified potential support delivery features that would be beneficial, including individual peer-to-peer support, group-based messaging, professional moderators, and discussion boards. Additionally, participants discussed considerations when choosing peer supporters, such as using the same technologies (eg, the same continuous glucose monitor).

#### Platform and Software Selection

Given the anticipated benefits of a flexible setup and the ability to customize the app, digital health software companies were first reviewed. These included Weltel, Latero Labs, Freshworks, Tactica Interactive, and Tech Samurais (currently Four Nine Digital). However, due to financial constraints prohibiting the development of a de novo app, we decided to develop the app by adapting and expanding existing communication–based software. Alternative solutions were researched to identify which would best fit the identified requirements while making only limited changes to the app.

Slack, Mattermost, and Rocket.Chat were identified as potential platforms (Table 1). Slack was disqualified due to a lack of options for modification given its closed-source software, leaving Mattermost and Rocket.Chat as the final candidates for consideration. Rocket.Chat was selected for its customization, comprehensive documentation, and polished user interface and user experience design. Subsequently, Rocket.Chat was tested with end user partners (n=11) to evaluate user experience before being modified to create the REACHOUT app.

**Table 1.** Alternative software options and criteria researched^a^.

Property	Slack [27]	Rocket.Chat [28]	Mattermost [29]
Customizability	Slightly customizable	Very customizable	Very customizable
Pricing (per user)	CAD $207/year; US $ ~145/year	Free if self-hosted or CAD $55/year; US $ ~38/year	Free if self-hosted or CAD $141/year; US $ ~99/year
Hosting costs	Included	CAD $2045/year; US $ ~1430/year	≤CAD $2100/year; US $ ~1469/year
Setup and maintenance	Easy and nontechnical	Complex	Complex
Hosting location	United States	Canada	Canada
Source	Closed-source	Open-source	Open-source

a Pricing in Canadian dollars reflects amounts at the time of inquiry. Conversion to US dollars was done on January 20, 2025.

#### Technology Stack and Platform Architecture

**Figure 3.**
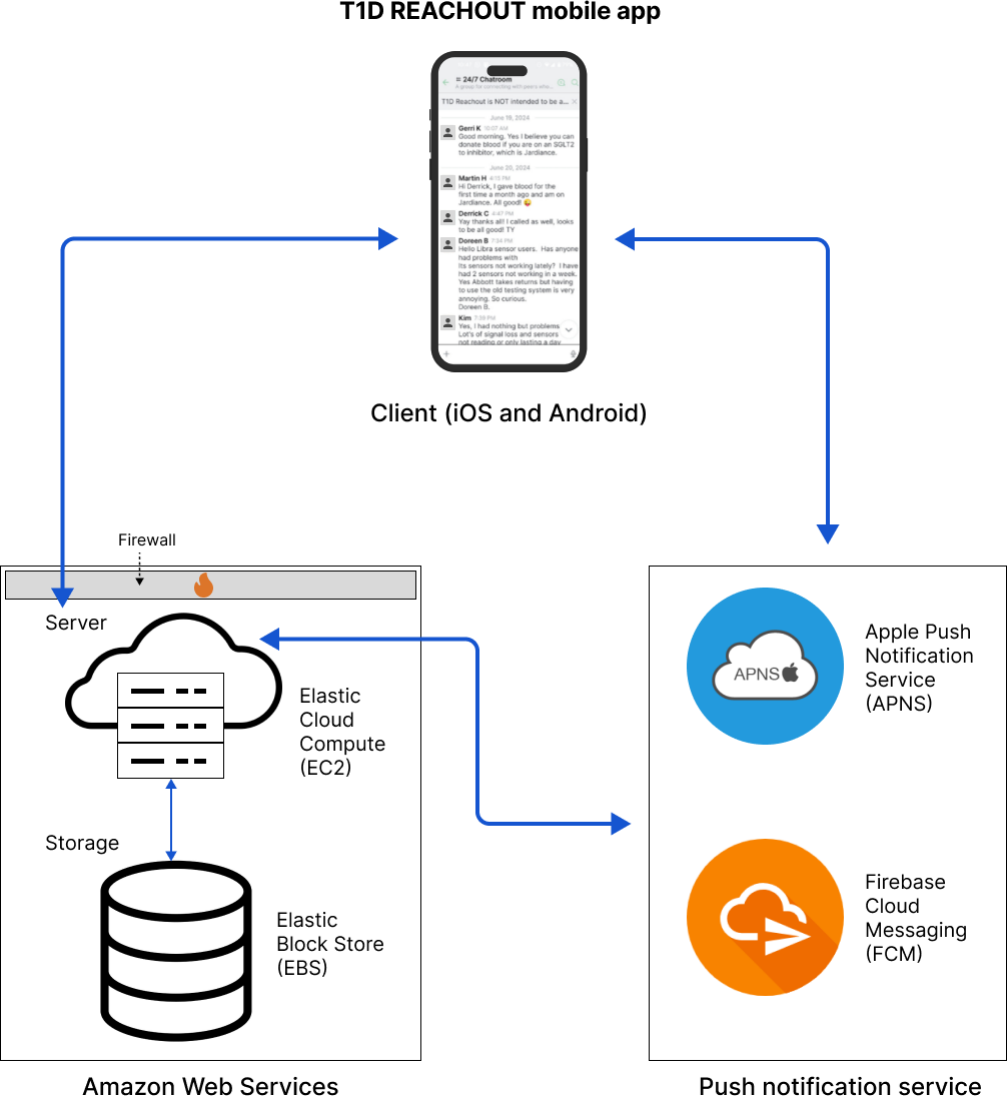
Overview of the current app architecture with interactions between client components, server and storage components, and third-party notification services. T1D: type 1 diabetes.

REACHOUT is built on the Rocket.Chat cross-platform messaging platform, powered by React Native. When new updates are made to the app, we build iOS and Android app files from the latest code changes using Xcode and Android Studio. The client-facing component includes an app for Android and iOS devices that interacts with our Amazon Web Services (AWS) backend via a firewall. An Amazon Elastic Cloud Compute instance provides compute capacity with Amazon Elastic Block Storage for long-term data storage. Initially hosted on DigitalOcean’s cloud, it was later migrated to a Canadian AWS instance at the recommendation of initial contractors and supported by more robust documentation from Rocket.Chat for AWS deployment. Push notifications use Apple Push Notification Service for iOS devices and Firebase Cloud Messaging for Android devices (Figure 3).

#### Focus Groups

Six focus groups with 38 participants (5‐7 participants per focus group) were held to identify users’ current and past T1D-related mental health needs, explore desired features for the app, elicit peer supporter communication mode preferences, and understand patterns of social media use for diabetes-specific support [1]. Detailed results have been reported previously [1].

Through these focus groups, participants established the following key requirements:

1. One-to-one support from a peer supporter (an individual with similar firsthand experience) could help users navigate the “ups and downs” of living with T1D.
2. 24/7 group support allows participants to receive support at any time of day, including evenings and weekends. It is difficult to predict when users need immediate emotional validation, normalization, advice, and acceptance. For example, participants cited hypoglycemia, which can occur in the middle of the night, as a situation in which immediate emotional support can provide reassurance and comfort.
3. Synchronous and asynchronous support options are available for meeting virtually with others or asking or responding to questions in participants’ own time.

Focus group participants also provided feedback on specific app features that would be beneficial, further informing development [1]. These recommendations included (1) which data to incorporate in peer supporter profiles, such as age of diagnosis, hobbies, stage of life, and T1D monitoring and treatment technologies used (eg, brand of continuous glucose monitor) so that participants can determine a suitable match; (2) having trained health professionals on the platform to prevent the exchange of medical information; (3) including resources and links to keep up with best practices; and (4) hosting topic-specific group discussions on subjects specific to living with T1D.

From focus group discussions, we identified 6 key features based on recurring needs and priorities articulated by participants and prioritized in development based on feasibility [1]: peer supporter profiles, a peer supporter library, one-to-one messaging, group messaging, topic-specific discussion boards, and virtual huddles.

### Stage 2: Co-Design and Development

#### Release and Feedback Cycle

An external developer was contracted to translate the user-identified key features into an initial prototype. Three iterations of the prototype were developed and released to subsequent groups of test users. Over the course of each iteration, users were asked to provide feedback to the research team via Zoom meetings regarding feature design preferences, functionality, and pain points (eg, a lack of intuitiveness in performing an action).

The first iteration (released to 23 users: 17 iOS users and 5 Android users) included peer supporter profiles, one-to-one messaging, and group messaging, which were more feasible to implement given Rocket.Chat’s existing functionality. Feedback was received and implemented in the following iteration. The second iteration (released to 43 users: 31 iOS users and 12 Android users) included the existing features and added video huddles. The third iteration retained the features we had already updated (peer supporter profiles, one-to-one messaging, and group messaging), updated the video huddles feature, and added a peer supporter library.

There was approximately 25% overlap in users across iterations, as users participated in consecutive app iterations. We sought to recruit 50 participants and recruited 46. Given that this was a feasibility study, no power calculation was required. To determine a reasonable sample size, we conducted a literature review and identified other pilot studies similar in topic. According to a systematic review of mHealth apps for chronic illness and/or social support, 12 of the 13 studies reported samples of fewer than 50 participants, with an age range of 12 to 49 years [30]. Considering these studies, we elected to recruit a sample size of 50 participants.

#### End User Partners

A small group of end user partners (n=11) was engaged to support iterative collaboration throughout development. End user partners contributed to wireframe development, design recommendations, and postimplementation feedback, such as ranking the importance of content on peer supporter profiles and rating the usefulness of proposed features. Feedback was elicited through 3 group meetings, a one-on-one phone call, and an email discussion. This enabled end users to influence design decisions and support iterative development grounded in lived experience.

The mean age of end user partners was 39 (SD 11) years; 55% (n=6) identified as female. The small number of end user partners was not intended to be representative of the T1D population as a whole but rather to support meaningful co-design through in-depth engagement.

### Stage 3: Deployment and Revision

#### Deployment

Following these 3 development iterations, the app was tested in a pilot study published elsewhere [2] and involved 46 users receiving support and 36 peer supporters delivering support. The mean age of users receiving support was 44 (SD 15) years, with the majority (n=29, 76%) identifying as women. The mean age of peer supporters was 41 (SD 16) years, with 37 (73%) identifying as women [12]. Of the 46 participants onboarded during app deployment, 8 withdrew for personal reasons. Of the 37 peer supporters onboarded, 2 withdrew, also for personal reasons.

We determined which participants were iOS or Android users and collected their respective App Store and Google Play Store email addresses. For iOS users, we used TestFlight to distribute the app builds. Users were provided access to a test version of the app. When new versions of the app were released, they were either automatically updated on the users’ mobile phones or could be downloaded manually via the TestFlight app. For Android users, we initially distributed an Android Package Kit file that users downloaded, but later pivoted to releases on the Google Play Store for ease of use. Users were sent a link to download the app, and any updates were automatically downloaded to their mobile phones. Only invited users could download the app for either Android or iOS.

#### Platform Modification Focus Groups

A further set of postpilot focus groups was held to receive feedback on user priorities for the app and potential modifications. Features that needed to be fixed and features to add based on user feedback were presented [31], and users (n=8) completed a prioritization exercise, classifying features into must have, should have, could have, and won’t have [32].

#### User Testing of Discussion Board and Home Page

**Figure 4.**
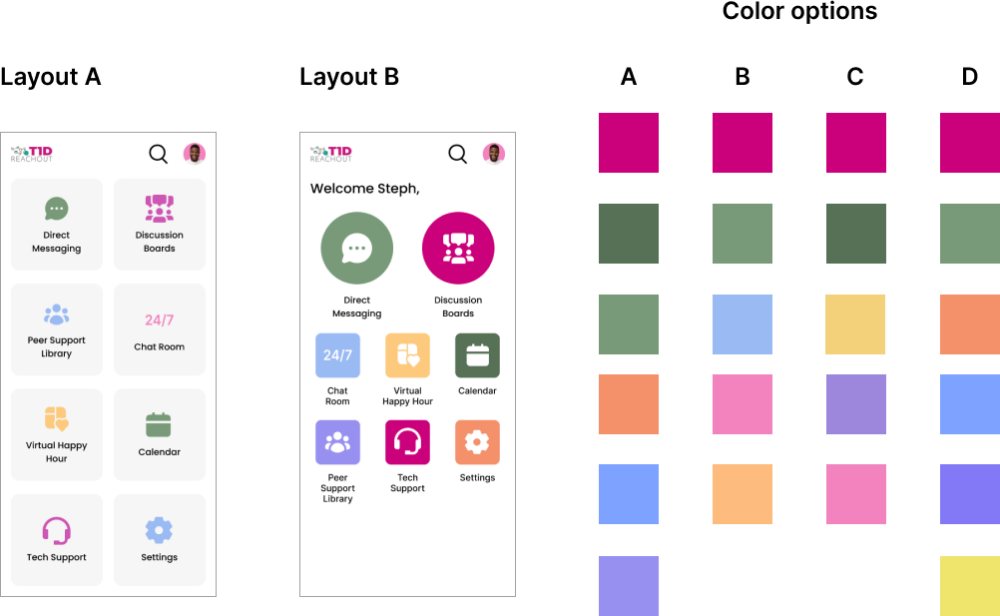
Home page user testing with 2 layout options and 4 color options. Layout A uses a card layout with larger icons, while layout B uses a grid layout with smaller icons. Both layouts were presented with each color option.

Given discussion boards as a high priority, user testing was conducted to determine user flows that support both anonymous and identifiable posts on a discussion board and to allow users to bookmark (save) posts for later reference. Feedback was also received on the design of the discussion board menu and the home page (Figure 4).

#### Further Postdeployment Development and Implementation

The new features and modifications identified through the postpilot focus groups and user testing were translated into wireframes by a contracted designer and then implemented by a contracted developer for the RCT of REACHOUT. Our platform team was required to fix bugs and defects internally and implement user-requested features that the developer had not delivered. These internal changes resulted in a 4-month delay in the launch of the REACHOUT app for the RCT.

## Platform Features

### Overview

The iteration of the REACHOUT app that was released for the RCT contains the following 6 key features identified in the initial stage 1 focus groups: a 24/7 community chat room (group text messaging), topic-specific discussion boards, a peer supporter library, peer supporter profiles, small group virtual sessions (ie, virtual happy hours), and direct (one-to-one) messaging. Each of these features is described below, along with an accompanying figure showing the progression of development based on user feedback, from earliest design (conception) to the final iteration used for the RCT.

### 24/7 Community Chat Room

**Figure 5.**
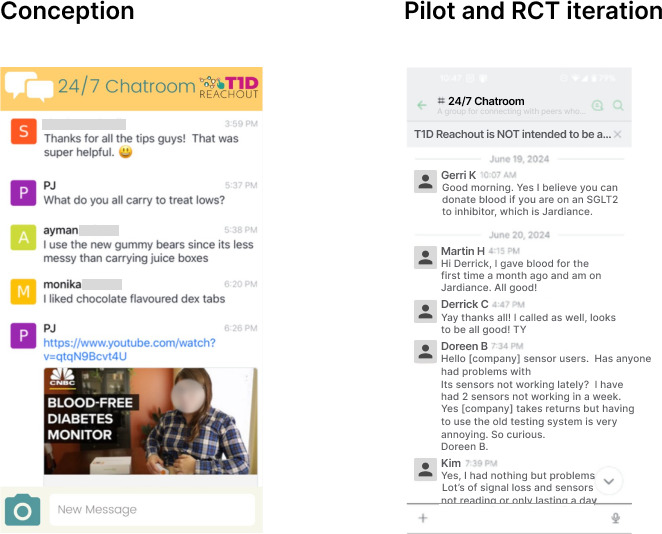
The concept and randomized controlled trial (RCT) interface of the 24/7 chatroom.

The 24/7 chat room is a feature built upon Rocket.Chat’s existing microservices architecture (Figure 5). Users can access a general-purpose discussion forum and send text, images, and voice messages. A banner reminds users of community rules, such as not providing peers with medical advice. In fact, group exchanges are monitored by research assistants and health care professionals (registered psychologist, dietitian, diabetes nurse, and pharmacist).

### Topic-Specific Discussion Boards

**Figure 6.**
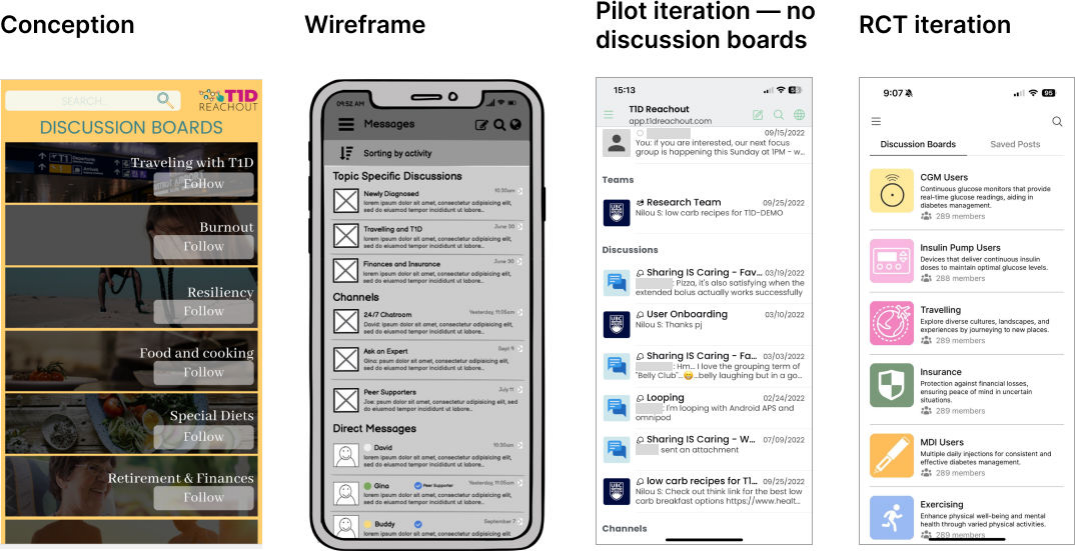
Discussion board iterations, including conception, wireframe, pilot iteration, and the randomized controlled trial (RCT) iteration, based on user feedback from the co-design and development process.

According to pilot study feedback, exchanges on the 24/7 chat room were perceived as overwhelming to some users, and message threads could easily be lost [31]. This formed a new requirement: creating topic-specific discussion boards for focused messaging channels limited to a specific T1D topic. These discussion boards were developed as separate public messaging channels using Rocket.Chat’s channel creation feature. The app was modified to create a designated menu page for topic-specific discussion boards. User-identified topics on current discussion boards include exercise, multiple daily injections, insulin pumps, insurance coverage, continuous glucose monitors, and traveling (Figure 6). The research team can add additional discussion boards, and frequently initiated topics will be retained for future platform instances.

### Peer Supporter Library

**Figure 7.**
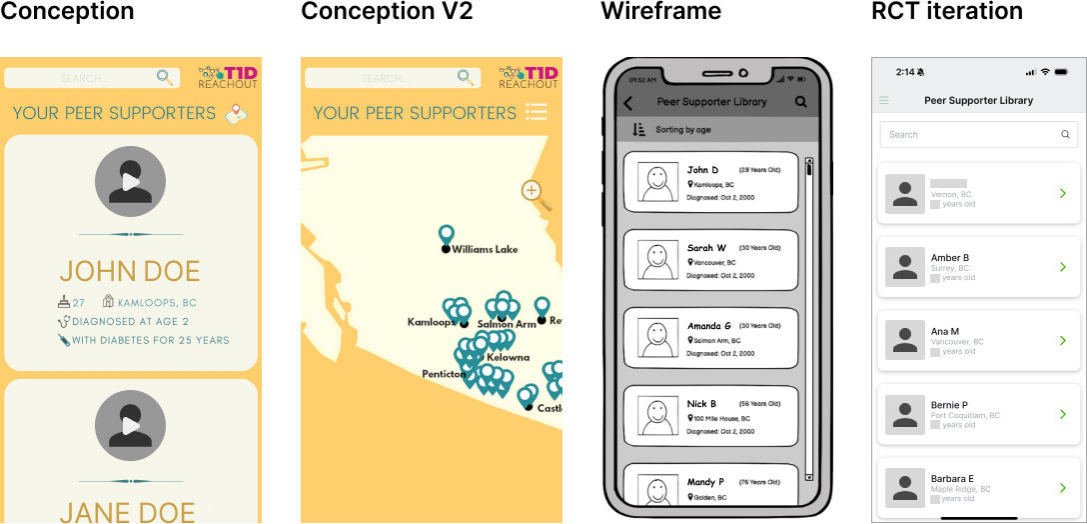
Peer supporter library iterations, including conception, a second conception with a location-based library, a wireframe, and the randomized controlled trial (RCT) iteration.

The peer supporter library invites participants to self-select a peer supporter [2]. The implementation of this feature was initially explored in two ways: (1) a participant (one who receives support) browses profiles from the library and then chooses their own peer supporter (active matching) or (2) a participant conveys their preferences to the research team, which assists with selection (passive matching). The active approach was adopted so that users could identify a peer supporter who best fits their unique support needs. For example, participants considering having children might prefer a peer supporter who is already a parent. Rocket.Chat’s mobile app was customized to add this peer supporter library view (Figure 7). The library previews the first name and last initial, location, and age displayed as a card, with access to review the complete peer supporter profile enabled by tapping each profile card.

### Peer Supporter Profile

**Figure 8.**
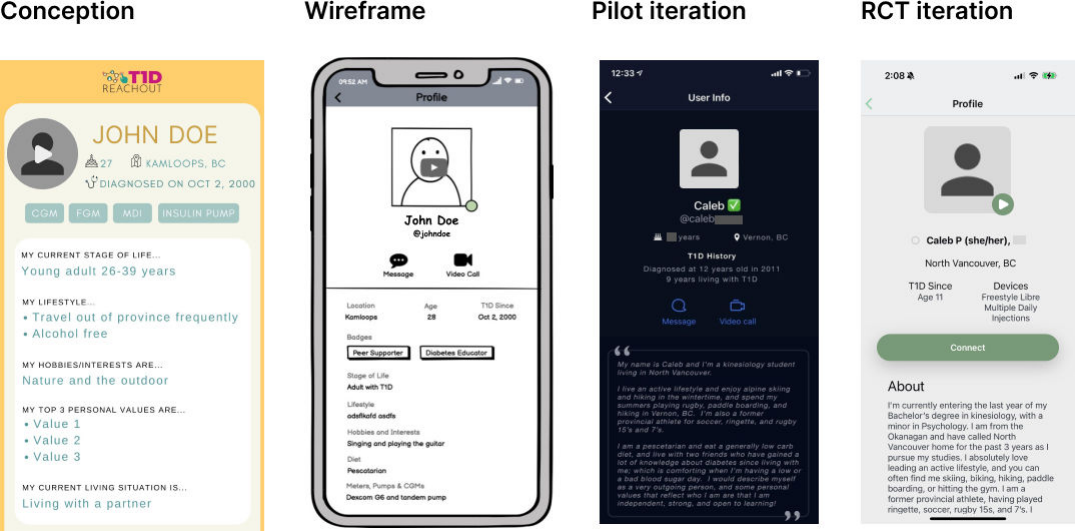
Peer supporter profile iterations, including conception, wireframe, pilot iteration in dark mode, and the randomized controlled trial (RCT) iteration.

To initiate an active matching approach, participants must determine which peer supporter best fits their support needs. To aid this, each peer supporter completes a profile that can be accessed through the peer supporter library [2]. The characteristics included in each profile (Figure 8) are first name and initial of last name, age, age at the time of diagnosis, and devices used (eg, type of insulin pump and continuous glucose monitor). Each profile also contains a written biography and a 1-minute video biography accessible via a private YouTube link [2].

### Small Group Virtual “Happy Hour” Sessions

“Happy hours” are virtual synchronous small group meetings led by peer supporters. They highlight distress-related themes, such as workplace bias and discrimination. Currently, information on virtual happy hours is shared via announcements posted to a discussion board, and sessions are held on Zoom (Zoom Video Communications Inc.). For the RCT, virtual happy hours replaced virtual huddles, featured in the previous pilot study [2].

### Home Page

**Figure 9.**
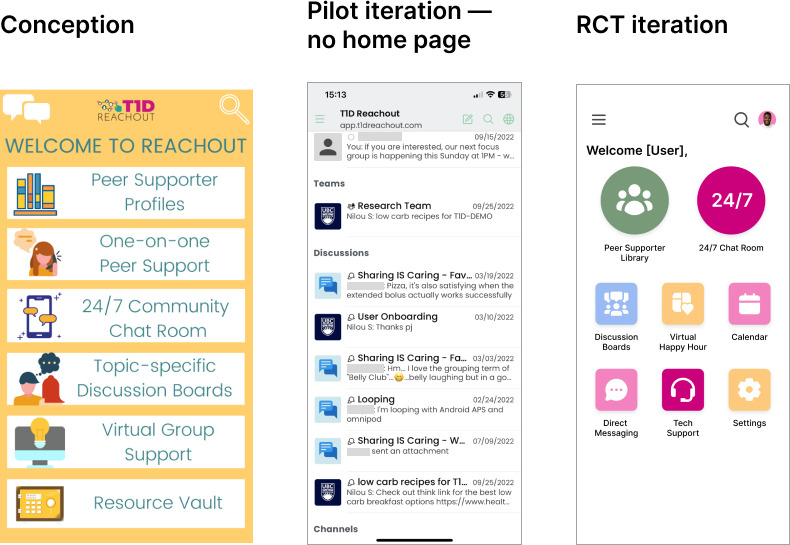
Home page iterations, including conception, pilot iteration without a home page (which directly jumped into the 24/7 chat room), and the randomized controlled trial (RCT) iteration after feedback from adolescent focus groups.

Adolescent focus groups identified a new requirement [33]: an enhanced user interface and user experience, achieved by making the home page less intimidating and more visually appealing. One example was a landing page shown when the app opened (Figure 9). The home page also functions as a directory where users can navigate to other app areas.

## Initial Platform Utilization Patterns

To analyze trends in this peer network intervention, timestamps of messages, along with their senders and recipients, were logged between October 12, 2021, and March 15, 2022. A network analysis was performed using NetworkX [34], and usage patterns were interpreted qualitatively by identifying patterns (numbers and widths) in edges, direction of communication, and temporal characteristics as the study progressed.

During a 6-month period when the app was deployed for the REACHOUT pilot study, 10,410 messages were sent, including 7116 one-to-one direct messages and 1389 chatroom messages; 179 private messaging groups were created. One-to-one messaging was the app’s most common mode of written communication and was a key requirement. The most one-to-one messages were between peer supporter-participant pairs (3446), followed by 802 between peer supporters and health care team members. Users explained that group messaging, such as the 24/7 chatroom, was more commonly browsed than contributed to.

**Figure 10.**
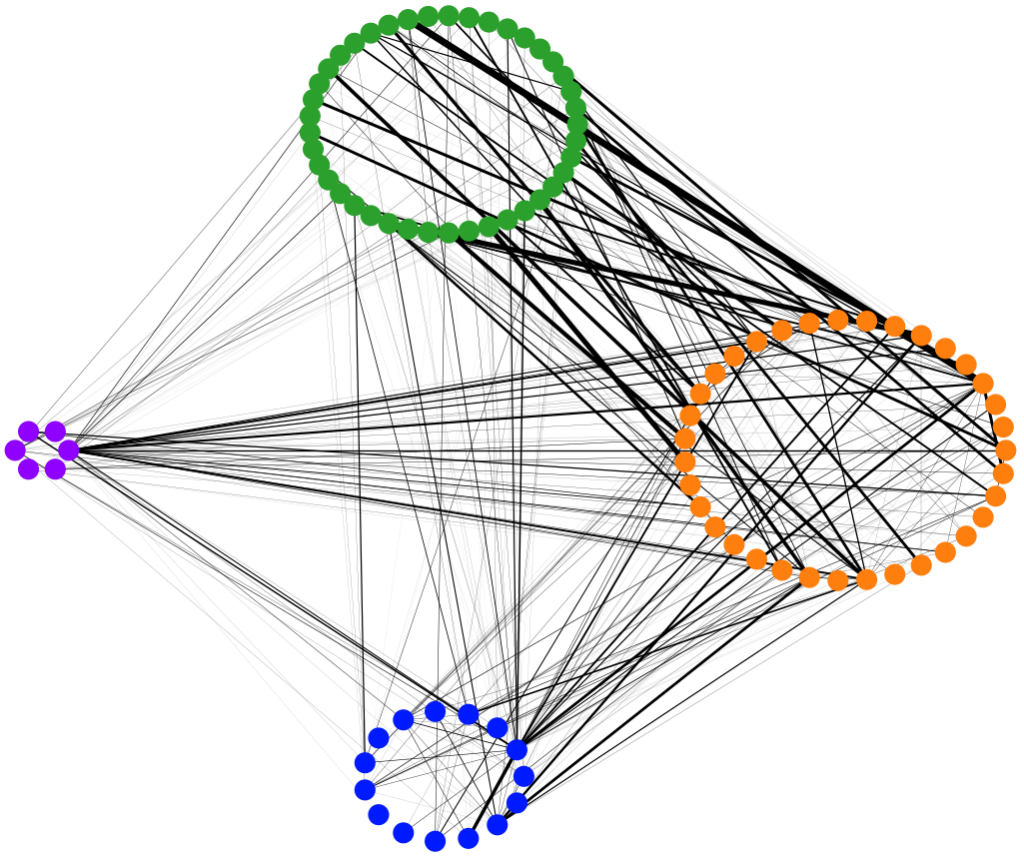
Network analysis showing communication patterns among app users by user type, specifically the defined roles assigned to users within the app (blue: research team, purple: health care team, orange: peer supporters, and green: participants). The number of messages sent is represented by the line width (edge weight), while the directionality of messages is not considered in this graph.

Substantial communication occurred between participants and their self-selected peer supporters (Figure 10), likely driven by an intervention requirement to engage with their assigned participants at least weekly (later changed to every 2 weeks based on feedback). This illustrates the app being used as intended, that is, as a mechanism to deliver peer-led mental health support primarily between a participant and their self-selected peer supporter. A total of 3446 messages were exchanged between participants and their self-selected peer supporters. However, communication methods varied among peer supporter-participant pairs, with some preferring Zoom or phone calls, which were not captured in the network graphs. Frequent communication also appeared to occur between peer supporters themselves and between peer supporters and the health care team.

## Insights and Observations: Challenges to Co-Design in mHealth

Through an iterative co-design process, we learned the importance of end-to-end user engagement to improve the user experience and ensure users’ needs are met. This collaboration refined the features developed from early iterations (eg, user-identified requirements for one-to-one support via direct messaging and group support in the 24/7 chatroom) into later iterations (eg, developing topic-specific discussion boards based on user feedback). Yet, the process was also challenging, particularly in establishing consensus on requirements across several focus groups, and when users’ stated wants did not align with what they needed [35].

The focus groups used in the app design process varied in size—this meant that the needs of 1 group were not necessarily representative of all users’ needs. We would seek input from different groups and, understandably, receive different opinions from each. It may have been beneficial to have a process to make the final call in these scenarios. We attempted strategies, such as voting or “buying” features, but ultimately, not everyone would be satisfied, as users will necessarily have unique needs.

While we had a general understanding of how features should function, the design and functional requirements were often not communicated clearly between the designer and the developer. Consequently, the delivered features sometimes differed from the research team’s expectations. For example, the home page design was not implemented as displayed in the mock-up. We sought to resolve this by adding wireframes and gathering feedback from the team or participants earlier. However, frequently incorporating participant feedback to enhance the user experience sometimes led to revising features already developed or in progress, which hindered our progress.

Given that Rocket.Chat is a large open-source codebase with the React Native repository currently having 131 contributors, it took new developers significant time to onboard and become familiar with the codebase, which also led to delays. Furthermore, because our resources only allowed for a single contracted developer working part-time (20 hours per week), always remotely, and sometimes in different time zones, it took substantial effort to receive deliverables on time.

To mitigate these challenges, we took a hybrid approach following the pilot study for subsequent development and maintenance of the REACHOUT app by hiring a vendor to handle the development of complicated and time-consuming tasks, which were of lower priority, and an in-house product manager with technical expertise to manage the execution of the product development and budgetary management, proper communication of requirements to the vendor, biweekly release of the app for internal testing, and continuous assessment of the work performed by the vendor to resolve issues promptly. We also had internal student developers with a background in computer science on the research team that worked on urgent features, minor improvements, bug fixes, and internal app testing. This approach ensured improved coordination, task prioritization, project management, efficient development and quality control, and better documentation, helping eliminate skill gaps. Though we acknowledge that there are vendors who offer quality service at a reasonable cost and other vendors who are expensive, but take care with proper coordination, communication, management, design, development, and testing to ensure a quality product is delivered to their customers, we found our hybrid approach to be sustainable and budget-friendly compared to fully internal or fully external development, allowing us to release a stable and reliable version of the app to our users in a reasonable timeline.

## Discussion

### Overview of Findings

In collaboration with adults from the T1D community in British Columbia, we designed and implemented REACHOUT, a mobile app for adults with T1D in British Columbia, which seeks to reduce diabetes distress (a core facet of diabetes-specific mental health). This project builds upon previous work establishing virtual peer support as a viable strategy for reducing diabetes distress [1,13]. Six key user-defined features for the app were identified through focus groups and refined through co-design and a pilot deployment: peer supporter profiles, a peer supporter library, one-to-one messaging, group messaging, topic-specific discussion boards, and small group virtual sessions.

The REACHOUT app uniquely features a user-driven “match-making” process to access one-on-one peer support [2]. Specifically, each user chooses their peer supporter based on the factors that matter most to them (eg, shared hobbies, lifestyle, insulin pump and/or continuous glucose monitor brand, location of residence) rather than the selection determined by the research team or those administering the app. An iterative co-design approach enabled early and comprehensive user engagement, identified app requirements, enabled prompt identification of pain points (eg, usability issues and bugs), and provided us with ongoing formative feedback from end users.

### Limitations

Our app development process had its limitations. First, no formal usability testing was undertaken because (1) the importance of such testing was not initially identified and (2) the tight timelines and constrained resources of our behavioral research study were challenging. Formal usability testing using standardized questionnaires, such as the System Usability Scale [36], Questionnaire for User Interaction Satisfaction [37], or Post-Study System Usability Questionnaire [38], could have ensured that users were sufficiently able to use the app and contribute to our overall understanding of feasibility. While the lack of formal usability testing may have affected the app’s quality, user feedback from focus groups was incorporated before the app was used in the RCT. Additionally, focus group participants were predominantly female and self-selected, which may not reflect the larger T1D community in British Columbia. Future work should ensure diverse voices are represented when establishing app requirements, for example, by increasing the number of male participants.

### Future Work

The app has been deployed and is being evaluated in the REACHOUT RCT (NCT05668507). A formal usability evaluation should be performed to examine the app’s functionality and identify opportunities for additional features or device optimization. Features currently in development, based on further user feedback, include allowing peer supporters to limit the number of participants they connect with. When evaluating usability, participants’ usage practices, motivations, and intents should also be examined to understand ideal usage patterns. We are also codeveloping an adolescent-focused version of the app [33], which considers adolescents’ unique support needs and will be evaluated in a future trial.

### Conclusions

The REACHOUT app was developed to reduce diabetes distress by providing peer-led mental health support to adults living with T1D in British Columbia. The app development process was described to guide others on similar projects. An iterative co-design process with end-to-end user engagement and continual user feedback was beneficial, enabling a thorough understanding of end user needs, preferences, and context, and ensuring the final app was impactful and relevant to end users. The REACHOUT app offers choice-based, just-in-time, and customizable support, making it the optimal peer support model for individuals of all socioeconomic backgrounds, geographic residences, and life circumstances [2]. Its potential to reduce diabetes distress and improve diabetes-specific mental health outcomes is being evaluated in a large-scale province-wide RCT.

## Abbreviations

AWS: Amazon Web Services
mHealth: mobile health
RCT: randomized controlled trial
T1D: type 1 diabetes
